# Mitochondrial respiratory complex I proteostasis: The role of a subunit turnover rate

**DOI:** 10.1093/plcell/koad149

**Published:** 2023-05-23

**Authors:** Lucas Frungillo

**Affiliations:** Assistant Features Editor, The Plant Cell, American Society of Plant Biologists, USA; Institute of Molecular Plant Sciences, School of Biological Sciences, University of Edinburgh, UK

The mitochondrial respiratory Complex I (CI) catalyzes the first step in the electron transport chain and contributes to generating the proton gradient across membranes required for ATP synthesis (Maldonado et al. 2020). Intrinsic to its enzymatic activity and subcellular environment, CI is prone to oxidative damage and displays a relatively high turnover rate. Despite its importance in bioenergetics, the control of CI stability and activity is not fully understood. In this issue of *The Plant Cell*, **Abi S. Ghifari and colleagues (Ghifari et al. 2023)** investigate the mechanisms controlling CI proteostasis and show that a mitochondrial chaperone facilitates complex disassembly and turnover. This work sheds light on long-standing questions about the mechanisms controlling CI homeostasis and demonstrates how protein complex activity is tuned by the turnover of individual subunits.

The mitochondrial CI ASSEMBLY FACTOR 1 (CIAF1) facilitates iron-sulfur (Fe-S) cluster insertion into the matrix subunits of CI (Ivanova et al. 2019). Arabidopsis *ciaf1* knockout plants display dwarfism due to deficient CI assembly. To identify novel mechanisms controlling CI proteostasis, the authors visually screened an ethyl methane sulfonate–mutagenized *ciaf1* population for growth recovery. Whole-genome sequencing of selected ethyl methane sulfonate–mutagenized plants showed that a serine to phenylalanine amino acid change at position 70 (S70F) in NADH DEHYDROGENASE UBIQUINONE IRON-SULFUR PROTEIN 7 (NDUFS7, also called PSST) rescued the growth phenotype of the *ciaf1* mutant. PSST, a 20-kD subunit located at the matrix domain of CI, is involved in the ubiquinone binding and reduction steps at CI (Soufari et al. 2020). Blue-native polyacrylamide gel electrophoresis, followed by in-gel NADH dehydrogenase activity assay, revealed that *psst ciaf1* (also referred to as *rmb2 ciaf1*) double mutants displayed increased CI abundance and activity compared to *ciaf1*. High-resolution isoelectric focusing liquid chromatography-mass spectrometry proteomics revealed significantly higher levels of CI matrix subunits in *psst ciaf1* plants compared to *ciaf1*. Together, these data show that PSST^S70F^ elevates CI levels and activity in the CI-deficient *ciaf1* mutant.

Next, the authors sought to investigate the underlying molecular mechanisms by which PSST controls CI proteostasis. Analysis of degradation rates of CI subunits by ^15^N labelling followed by quadrupole time-of-flight mass spectrometry of soluble matrix fractions showed overall slower turnover rates for subunits located within the CI NADH-ubiquinone-binding modules, but not for other oxidative phosphorylation subunits, in *psst ciaf1* compared to *ciaf1*. These data suggest that S70F in PSST is required for CI subunit turnover. Previously, the authors had shown that the ATPase, but not the proteolytic activity, of the mitochondrial AAA + metalloprotease FILAMENTOUS TEMPERATURE SENSITIVE H3 (FTSH3) impacts CI abundance and activity in plants (Ivanova et al. 2021). Confirming previous findings, mass spectrometric analysis of peptides co-immunoprecipitated with the CI subunit B14.7-FLAG identified FTSH3. The authors then hypothesized that FTSH3 directly interacts with PSST to control CI subunit turnover. Indeed, yeast 2-hybrid and bimolecular fluorescence complementation assays revealed that FTSH3 interacts in vitro and in vivo with PSST but not with PSST^S70F^ (Fig.). Homology modeling of PSST suggested that the bulky benzyl group of phenylalanine in PSST^S70F^ leads to a larger helix size and disruption of intramolecular interactions. Based on the PSST protein model, the authors propose that the S70F mutation controls substrate recognition by altering PSST thermodynamic stability. Suppression of PSST–FTSH3 interaction slows the turnover of NADH-ubiquinone–binding modules and provides a framework to regulate CI activity in plants.

**Figure.**
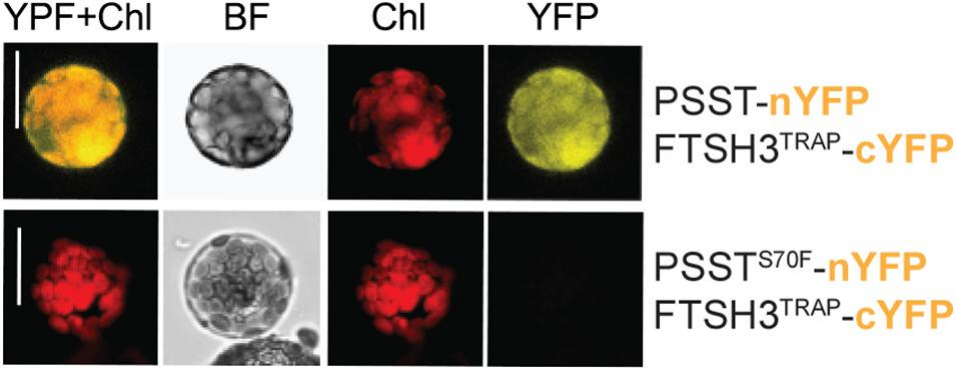
FTSH3 interacts with the CI matrix subunit PSST. Bimolecular fluorescence complementation assays of proteolytically inactive FTSH3^TRAP^ and PSST (with or without the S70F mutation) fused to either the C-terminal or the *N*-terminal half of the yellow fluorescent protein (cYFP/nYFP). YFP fluorescence, chlorophyll (Chl) fluorescence, the bright field (BF) image, and the overlay of YFP and chlorophyll (YFP + Chl) fluorescences are shown. Scale bar, 20 *μ*m. Adapted from Ghifari et al. (2023), Fig. 4C.

Looking forward, the authors plan to investigate the role of FTSH3 in regulating the homeostasis of other subunits of oxidative phosphorylation complexes. Because the FTSH family of metalloproteases contains multiple members, it is tempting to speculate that FTSHs fine-tune the activity of different mitochondrial complexes by a similar mechanism. Identifying the substrate specificities of other members of the FTSH family might prove fruitful in understanding how proteostasis of mitochondrial complexes regulates bioenergetics in response to environmental cues.
